# Intraoperative finding and management of complete spinal cord transection after thoracolumbar traumatic fracture-dislocation: A case report

**DOI:** 10.1097/MD.0000000000024096

**Published:** 2021-01-15

**Authors:** Dong-Ju Lim

**Affiliations:** Department of Orthopaedic Surgery, Seoul Spine Institute, Sanggyepaik Hospital, College of Medicine, Inje University, Seoul, Korea.

**Keywords:** case report, complete paraplegia, management of spinal cord, spinal cord complete transaction, traumatic spinal cord injury

## Abstract

**Rationale:**

We report the first case of the management of spinal cord transection due to thoracolumbar fracture-dislocation in human beings. There are several case reports of cord transection, but only radiological findings have been reported; we report intraoperative findings and management.

**Patient concerns:**

A 53-year-old man presented to the hospital after falling. He had no motor power or sensation below T10 (below the umbilicus area) dermatome level. American Spinal Injury Association (ASIA) impairment scale was grade A. Magnetic resonance imaging and computed tomography demonstrated a fracture and translation of the vertebral body at the T11-T12 level and anterior displacement of T11 on T12, with complete disruption of the spinal cord.

**Diagnosis:**

Complete spinal cord resection due to T11-T12 fracture-dislocation.

**Interventions:**

We performed spinal fusion with pedicle screw instrumentation (T10-L1) and autobone graft and decompression and repaired the dural sac to prevent cerebrospinal fluid leakage. There was no neurological recovery either immediately or 4 years post-operation at follow-up.

**Conclusion:**

To the best of our knowledge, this report is the first on the intraoperative finding and management of the complete transection of the spinal cord in thoracolumbar spine injury. Perfect fusion is required to facilitate rehabilitation and daily living, prevent neurogenesis, and prevent unnecessary pain such as phantom pain.

## 1 Introduction

Spinal cord injury occurs with injury to the vertebral column, producing mechanical compression or distortion of the spinal cord with secondary damage resulting from ischemic, inflammatory, and other mechanisms. Traumatic spinal cord injury (TSCI) is a problem that mostly affects young men as a consequence of motor vehicle accidents, falls, or violence.^[1]^ The neurologic injury classification tool produced by the American Spinal Injury Association (ASIA), the ASIA scale, classifies spinal cord injury according to the spinal cord level and the severity of neurologic deficits. Most TSCIs involve the cervical spinal cord and result in quadriparesis or quadriplegia. Suspected patients with TSCI with mental unconsciousness because of neurologic deficits and impaired alertness or potentially distracting systemic injuries require continued immobilization until radiologic studies exclude an unstable spine injury.^[2]^

Several cases of cord transection have been reported, but only radiological findings have been discussed. We report an intraoperative finding of complete spinal cord transection. Our report is the first of the management of spinal cord transection due to thoracolumbar (TL) fracture-dislocation in a human being.

This case report has been approved by institutional review board of Sanggyepaik Hospital, Inje University, who waived the need for obtaining informed consent (SGPAIK 2020-01-003).

## 2 Case report

### 2.1 Patient History, Presenting Features, and Radiological Investigation

**Figure 1:**
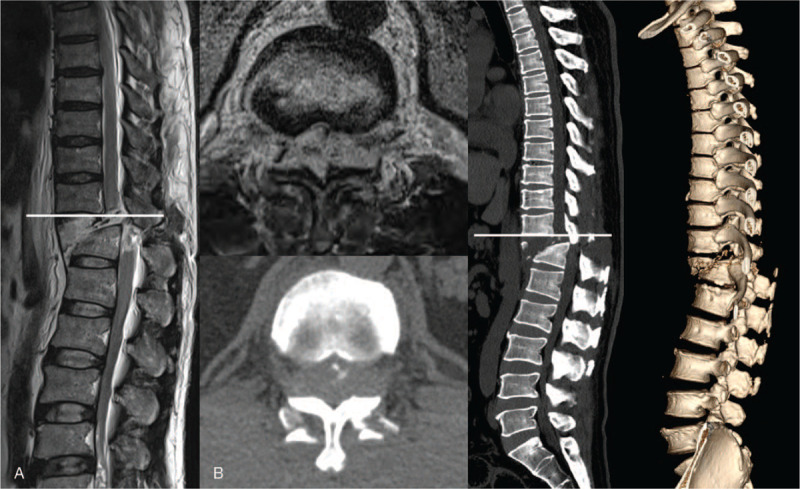
A. T2-weighted magnetic resonance imaging shows the dural defect. B. Three-dimensional computed tomography in the axial and reconstruction view shows fracture bone & fragment occupying the spinal canal.

A 53-year-old man was referred to the emergency department of our hospital after falling from 5 m at work. On physical examination, he had no motor power or sensation below T10 dermatome level (ASIA impairment grade A) and severe tenderness on the TL junctional area. There was no open external wound in the back, and sphincter tone disappeared. Magnetic resonance imaging (MRI) and computed tomography demonstrated a fracture and translation of the vertebral body at the T11-T12 level and anterior displacement of T11 on T12 (Figure 1). The diagnosis was complete transection of the spinal cord and T11-T12 fracture-dislocation. His vital signs were stable and he did not show symptoms of spinal shock.

### 2.2 Intraoperative Finding and Treatment

**Figure 2:**
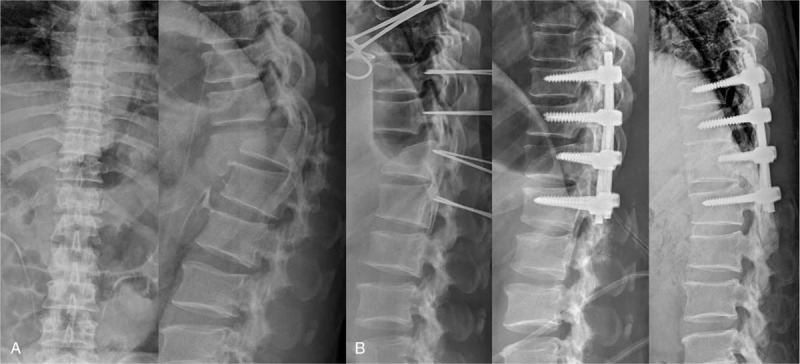
A. Anteroposterior and lateral radiograph of a patient presenting after injury with fracture-dislocation injury at T11/T12. B. Intraoperative K-wire X-ray and proper alignment of vertebrae immediately after the operation and at the 4-year follow-up.

**Figure 3:**
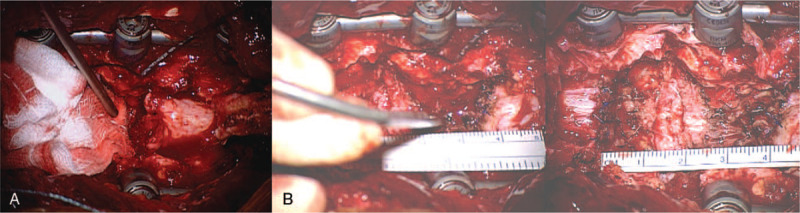
A. Intraoperative cord transection finding (blunt end margin). B. Dural end management with suturing in microscopic view.

During surgery, pedicle screws were inserted first, and then fracture alignment reduction was tried. However, we judged that fracture alignment reduction was not possible because of blocking facet, and then, both facets were removed. Following this, fracture reduction was performed at the site where the rod was implanted, and decompression performed. After permanent rod insertion, spinal alignment reduction was completed by the compression maneuver (Figure 2). The operator retracted both ends of the dural sac but did not pull. The size of the defect was about 4 cm in the microscopic field. We decided to perform suture because of the risk of cerebrospinal fluid (CSF) leakage expected during the patient's early rehabilitation and daily life activity. After irregular margins of the thecal sac were debrided, both ends of the spinal cord were sutured with prolene to avoid CSF leakage. Fibrin glue was applied, and CSF leakage was checked by the Valsalva maneuver test before closure (Figure 3).

### 2.3 Patient Prognosis and Rehabilitation

Postoperatively, neurological recovery did not occur. The patient was referred to an intensive care unit for monitoring and treatment of potential acute cardiovascular instability and respiratory failure. He applied compressive stockings to prevent embolic disease. On the first day post-operation, hemovac applied full negative pressure; after that, half negative pressure was applied. CSF leakage was not detected. One week after the operation, the patient began to sit with a thoraco-lumbar-sacral orthosis (TLSO). Rehabilitation started two weeks after surgery. There was no neurological recovery either immediately or 4 years post-operation at last follow-up. The patient has no pain on fracture-dislocation site during motion and daily activity through complete fusion. He had no symptomatic unexpected pain such as phantom pain.

## 3 Discussion

Traumatic spinal cord injuries, a component in multiple trauma, usually affect young people, are a significant cause of morbidity, and pose significant health care expenditures and considerable threats to survival and quality of life.^[3–5]^ Spinal cord injury affects mostly the cervical spine, whereas TL lesions are rare.^[1]^ In contrast to our report, a previous study reported females with a greater number of pre-existing co-morbidities, a higher frequency of TL trauma, and less severe neurological impairment.^[6]^

There have been many reports describing whether surgery can produce neurologic benefits. A significant cord transection can potentially contraindicate surgery.^[7]^ Moreover, without observing cord trauma, the dura may remain untouched in cord injury.^[8]^ Stauffer et al. reported no neurological benefits were found between surgical and non-surgical management of complete and incomplete spinal cord injuries.^[9]^ Moreover, defining surgical indications for closed TL fractures has been somewhat more challenging, in part because of difficulties in determining spinal instability in these lesions. Nevertheless, in many studies of patients with acute spinal cord injuries, rapid decompression and fixation resulted in good outcomes, and immediate surgery was determined.^[3,10–12]^ Management of delayed TL injuries is also challenging. The delay in presentation should not prevent spine surgeons from proceeding with operative intervention, as good results can be expected.^[13]^

Spinal cord transection refers to a tear within the spinal cord as a result of a significant traumatic injury. The degree of neurological compromise corresponds with the degree of cord transection. In a partial transection, there may still be some sensory-motor function retained, whereas in complete transection, there is a complete loss of function. Several cases have been reported of fracture or stab injury with spinal cord transection in humans, with minimal or no neurologic injury.^[14,15]^ Nonetheless, due to the limited ability of the central nervous system to repair itself following injury, many deficits remain permanent.^[16]^

Takahashi et al. reported a case in which duraplasty was performed. Neurologically, the sensory disturbance slightly improved 4 months after the injury.^[15]^ In our case, which presented cord parenchyma complete transection, the length of the dural tissue defect was about 4 cm, and there was no elasticity on both dural ends, so we could not directly repair it. Although spinal column shortening osteotomy could be applied to direct dural repair end-to-end anastomosis, osteotomy could not be performed due to the risk of massive bleeding and other organ damage immediately after acute high energy trauma.

Spinal cord transection is sometimes used for therapeutic purposes. In patients with scoliosis, it has been used therapeutically to restore bladder function.^[17]^ Lesions of the spinal cord dorsal root entry zone have been surgically created to relieve central pain because of spinal cord injury below-level neuropathic pain.

There have been many spinal cord experiments with rodents.^[18]^ However, rodents differ significantly from humans in anatomy, body size, and response to spinal cord injury.^[19]^ Results of spinal cord repair or regeneration are still poor. But in another experimental study, spinal cord tissue did not lose regenerative capacity, even after repeated transection, in the lamprey.^[20]^ Because of recent improvements in commonly used models and the development of new biology and technology paradigms, much progress is anticipated in the future.^[16,21]^

CSF leakage after spinal cord injury is relatively higher than that following cervical spinal surgery.^[22]^ On the other hand, the incidence of CSF leaks after surgery for cervical spinal trauma is relatively higher than that in cervical spinal stenosis. Therefore, 1 should expect the possibility of a dural tear and have a simple and effective management protocol for CSF leaks in trauma cases established.^[23]^ Reliable repair of the dura is needed to prevent possible surgical site infection and meningitis.^[24]^ In TL burst fractures with vertical laminar fractures, patients with wider interpedicular distance and larger encroachment fragments in the spinal canal were more likely to have dural tears.^[25]^

Treatment of patients with suspected traumatic spinal cord injury is also challenging because of neurologic deficits, and impaired alertness or potentially distracting systemic injuries that require continued immobilization until imaging studies exclude an unstable spine injury.^[2]^ This essential radiological finding can influence the decision on potential surgery in the setting of spinal trauma.^[7]^ MRI is the first choice evaluation tool and is often performed in recent traumatic spinal injury, which may or may not be confirmed by urgent computed tomography.

The role of urgent MRI is usually to assess for the presence of 2 treatable pathologies, cord compression or epidural hematoma, which can be targeted by surgical decompression. An initial poor neurological status of patients and disruption of the ligamentum flavum on MRI were predictable factors of dural tears and CSF leaks.^[22]^

## 4 Conclusion

To the best of our knowledge, this report is the first of the intraoperative finding and management of the complete transection of the spinal cord in TL spine injury. Perfect fusion is required to facilitate rehabilitation and daily living, prevent neurogenesis, and prevent unnecessary pain such as phantom pain. Surgical treatment is performed to avoid pseudomeningocele, CSF leakage, and infection. MRI is indispensable for accurate identification of the spinal cord injury before surgical treatment.

## Author contributions

**Conceptualization:** Dong-Ju Lim.

**Visualization:** Dong-Ju Lim.

**Writing – original draft:** Dong-Ju Lim.

**Writing – review & editing:** Dong-Ju Lim.

## Abbreviations

Abbreviations: MRI = magnetic resonance imaging, TL = thoracolumbar, TSCI = traumatic spinal cord injury.
